# Aquaporin-like water transport in nanoporous crystalline layered carbon nitride

**DOI:** 10.1126/sciadv.abb6011

**Published:** 2020-09-25

**Authors:** Fabrizia Foglia, Adam J. Clancy, Jasper Berry-Gair, Karolina Lisowska, Martin C. Wilding, Theo M. Suter, Thomas S. Miller, Keenan Smith, Franz Demmel, Markus Appel, Victoria García Sakai, Andrea Sella, Christopher A. Howard, Madhusudan Tyagi, Furio Corà, Paul F. McMillan

**Affiliations:** 1 Department of Chemistry, Christopher Ingold Laboratory, University College London, 20 Gordon St., London WC1H 0AJ, UK.; 2 University of Manchester at Harwell, Harwell Science and Innovation Campus, Didcot, Oxfordshire, OX11 0DE, UK.; 3 Electrochemical Innovation Lab, Department of Chemical Engineering, University College London, Torrington Place, London WC1E 7JE, UK.; 4 ISIS Neutron and Muon Source, Rutherford Appleton Laboratory, Harwell Science and Innovation Campus, Chilton OX11 0QX, UK.; 5 Institut Laue Langevin, 71 avenue des Martyrs, CS 20156, 38042 Grenoble CEDEX 9, France.; 6 Department of Physics and Astronomy, University College London, London WC1E 6BT, UK.; 7 NIST Center for Neutron Research (NCNR), National Institute of Standards and Technology, Gaithersburg, MD 20899, USA.; 8 Department of Materials Science and Engineering, University of Maryland, College Park, MD 20742, USA.

## Abstract

Designing next-generation fuel cell and filtration devices requires the development of nanoporous materials that allow rapid and reversible uptake and directed transport of water molecules. Here, we combine neutron spectroscopy and first-principles calculations to demonstrate rapid transport of molecular H_2_O through nanometer-sized voids ordered within the layers of crystalline carbon nitride with a polytriazine imide structure. The transport mechanism involves a sequence of molecular orientation reversals directed by hydrogen-bonding interactions as the neutral molecules traverse the interlayer gap and pass through the intralayer voids that show similarities with the transport of water through transmembrane aquaporin channels in biological systems. The results suggest that nanoporous layered carbon nitrides can be useful for developing high-performance membranes.

## Teaser

Water transport through aligned nanopores in layered carbon nitride is choreographed by H bonding similar to aquaporin channels.

## INTRODUCTION

Identifying materials that efficiently and selectively transport molecular water through artificial membrane structures presents a main challenge for designing next-generation water filtration devices and fuel cells (*1*). In biological systems, cells adjust and maintain their internal hydration levels while preserving the intracellular pH and ionic balance by means of aquaporins (AQP), which form tubular protein complexes embedded within the phospholipid bilayer cellular envelope. As H_2_O molecules travel in single file through the AQP central channel region, they reverse their orientation due to H-bonding interactions with amino acid residues lining the interior, thus ensuring the integrity of the transported molecules (*2*–*3*). This biological mechanism offers useful lessons for developing smart materials for synthetic membrane structures (*4*). Promising strategies are being developed using carbon-based materials (*5*–*6*). Although the oxidized form of graphite undergoes intercalation by water molecules, its narrow interlayer spacing does not permit long-range translational diffusion (*7*). Grafting ionic species within the layers allows tuning of the interlayer spacing and permits selective interlayer transport in graphene oxide (GO) for sieving applications (*8*). Thin-film laminates constructed from few-layered GO show selective water transport normal to the graphitic planes through nanopores occurring at interfaces between the stacked graphene domains (*9*–*12*). Similar performance has been noted for water permeation through graphitic carbon nitride (GCN) assemblies with intercalated anions used to control the interlayer spacing (*13*). However, although published illustrations often presume that H_2_O molecules travel single file in a "conga line" pattern through GO membrane assemblies, molecular dynamics simulations reveal evidence for the existence of bulk fluid regions trapped among the graphitic sheets that are involved in the flow process (*10*). Rapid water transport that more closely resembles the directional transport of single H_2_O molecules through AQP channels has been reported within subnanometer-diameter carbon nanotubes (CNTs) embedded in lipid membranes (*14*–*17*). Here, we report diffusion of single H_2_O molecules on a time scale equal to that in AQP channels across the interlayer spacing and through the aligned C_12_N_12_H_3_ ring pores within the stacked graphene-like layers of crystalline carbon nitride with a polytriazine imide (PTI) structure (*18*–*20*). The molecules undergo a sequence of orientation reversals choreographed by H-bonding interactions with ─N═ and ─NH═ species surrounding the ring voids within the carbon nitride layers to provide a transport mechanism that resembles that of H_2_O molecules transiting through biological AQP channels.

## RESULTS

### Synthesis and characterization of intercalant-free (IF-)–PTI and PTI·H_2_O

**Fig. 1:**
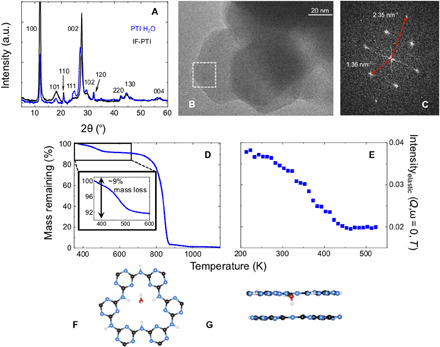
Characterization of IF-PTI and PTI·H_2_O materials. (**A**) Powder XRD patterns for IF-PTI and PTI·H_2_O. a.u., arbitrary units. (**B**) TEM image of hexagonal crystallites of IF-PTI showing lattice planes. (**C**) Fourier-transformed pattern showing characteristic diffraction spots of [110] (inner ring of spots in a hexagonal pattern) and [100] in-plane reflections of the PTI layers corresponding to real-space interatomic separations of 4.25 and 7.35 Å, respectively. (**D**) Thermogravimetric analysis (TGA) data for PTI·H_2_O between 300 and 1200 K. The initial reduction in mass of ~9% between 300 and 500 K is due to deintercalation of one H_2_O molecule per C_6_N_9_H_3_ formula unit of the layered PTI structure (*19*). The larger mass loss observed above 800 K is due to thermal decomposition of the IF-PTI carbon nitride phase. (**E**) Variation in elastic neutron scattering intensity observed during heating PTI·H_2_O between 200 and 530 K. (**F** and **G**) Top and side views of the C_12_N_12_H_3_ intralayer pores showing the equilibrium location of H_2_O molecules determined by DFT calculations.

Our crystalline PTI starting materials for this study were obtained by reacting dicyandiamide in eutectic LiCl/KCl or LiBr/KBr molten salt mixtures. These layered structures contained Li^+^ and Cl^−^ or Br^−^ ions intercalated between the stacked carbon nitride sheets or within the C_12_N_12_ rings, partially replacing H^+^ in the case of Li^+^ ions (*18*–*20*). Samples of intercalant-free IF-PTI were then produced by extracting the included ions into water followed by drying (*19*). Chemical analysis results were consistent with a layer composition C_6_N_9_H_3_ (table S1). X-ray diffraction (XRD) and transmission electron microscopy (TEM) combined with spectroscopic investigations showed that the crystalline structures and external morphology remained unchanged during the deintercalation process (Fig. 1, A to C). Upon exposure of fully dried IF-PTI to air, the samples spontaneously absorbed atmospheric moisture over a period of a few seconds to several minutes to produce the new intercalated compound PTI·H_2_O (*19*). Thermogravimetric analysis (TGA) of PTI·H_2_O indicated an initial mass loss of ~9 weight % during heating between 300 and 600 K corresponding to a single H_2_O molecule incorporated in each of the interlayer sites defined between the C_12_N_12_H_3_ rings in the PTI layers (Fig. 1D). The weight loss curve measured by TGA was mirrored by the decrease in neutron elastic scattering intensity observed on heating above 300 K (Fig. 1E). Our density functional theory (DFT) calculations described below show that in their equilibrium geometry, the H_2_O molecules are positioned relative to the intralayer voids by H-bonding interactions with ─N═ and ─NH─ groups that decorate the interior of the C_12_N_12_H_3_ rings (Fig. 1, F and G).

### QENS analysis of water dynamics in PTI·H_2_O

**Fig. 2:**
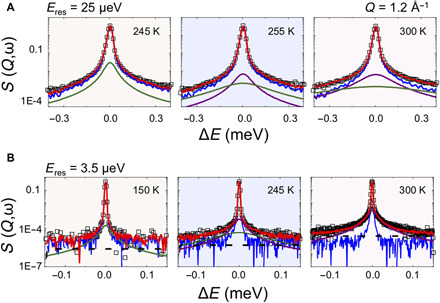
QENS analysis of PTI·H_2_O. (**A** and **B**) QENS data and their analyses shown at *Q* = 1.2 Å^−1^. Top panels were obtained at OSIRIS, ISIS, UK (*E*_res_ = 25 μeV), and the lower panel data were recorded at IN16B-BATS, ILL, Grenoble, France (*E*_res_ = 3.5 μeV). The global fit (red continuous curve) is overlain on the data points (black). Lorentzian fits to the QENS data after removal of the instrumental resolution profile convoluted with the elastic δ(ω) function are shown as continuous curves. Localized nanoconfined librational motions observed at 150 K in the high-resolution IN16B data are indicated by the green curve. The background function recorded at 5 K is shown underneath in blue. Localized dynamics are also detected in the OSIRIS data at 245 K. Above 255 K, *c*-*o*-*m* translational diffusion is observed in the OSIRIS QENS analysis (magenta), along with a broader contribution from pseudo-rotational/localized relaxation contributions (green curve). Additional *S*(*Q*,ω) datasets from the different instruments are shown in fig. S1.

**Fig. 3:**
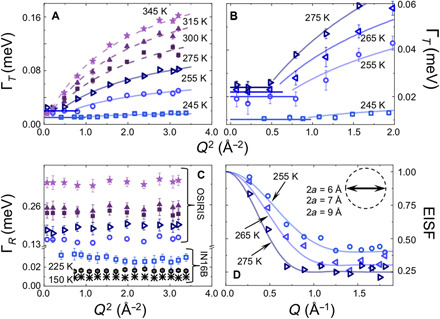
Diffusional and nanoconfined dynamics in in PTI·H_2_O. (**A**) Γ*_T_* versus *Q*^2^ plot showing evidence for *c*-*o*-*m* diffusional displacements of H_2_O molecules from QENS data above 255 K. A small *Q* dependence is also apparent in IN16B data at 245 K. Γ*_T_* (square symbols) is obtained as the half-height linewidth of the narrow Lorentzian component. (**B**) Detail of Γ*_T_*(*Q*^2^) for 245 to 275 K experiments showing the initial *Q*-independent signature of nanoconfined H_2_O dynamics. (**C**) Temperature behavior of the *Q*-independent Γ*_R_* contribution to the broad Lorentzian component in Fig. 2 (A and B). (**D**) EISF data obtained at OSIRIS and modeled by assuming rotation of intercalated water molecules nanoconfined within a spherical volume with radius *a*. The data were fit using 2*a* = 6, 7, and 9 Å at 255, 265, and 275 K, respectively (inset). A similar range of values was also estimated independently from fitting the plateau in the low-*Q* Γ*_T_* (*Q*^2^) data (Fig. 3A). The EISF for PTI·H_2_O above 300 K was modeled assuming free rotation of H_2_O molecules within a sphere of radius 0.98 Å constrained by the O─H bond length.

We studied the dynamics of water in PTI·H_2_O at temperatures between 150 and 345 K using quasi-elastic neutron scattering (QENS) that examines Lorentzian broadening occurring around the base of the elastic peak due to diffusionally mobile or nanoconfined motions of H_2_O molecules (Fig. 2, A and B). Our study used instruments at several facilities to achieve energy resolutions (*E*_res_) designed to probe the different relaxation processes and disentangle various contributions to the water dynamics over time scales extending from a few tens of picoseconds to several nanoseconds. We began by examining the fully dried IF-PTI sample. No Lorentzian broadening could be detected at any temperature, even in data obtained with the smallest instrumental resolution [*E*_res_ = 1 μeV at National Institute of Standards and Technology (NIST), USA; fig. S1]. That result indicated that bound N-H protons only undergo femtosecond vibrational excitations much faster than the observational time scale and that contributed to the flat background observed in all the QENS results for PTI·H_2_O samples. In contrast, the PTI·H_2_O samples revealed QENS dynamics at all probe time scales, indicating the presence of motional relaxations occurring on picosecond-nanosecond time scales. The QENS profiles were analyzed in terms of one or two Lorentzian functions, as required to fit the measured datasets (Fig. 2, A and B). QENS data are interpreted by examining the behavior of the half-width at half-maximum (HWHM; Γ) corresponding to the inverse of the relaxation time scale in relation to the momentum transfer *Q* (Fig. 3, A to D). Relaxation dynamics exhibiting a dispersive Γ*_T_*(*Q*^2^) relation correspond to translationally mobile water molecules with center-of-mass (*c*-*o*-*m*) displacements extending over length scales up to ~40 Å (Fig. 3A), whereas nondispersive Γ(*Q*^2^) dynamics indicate the presence of locally nanoconfined motions (Fig. 3, A to D).

**Fig. 4:**
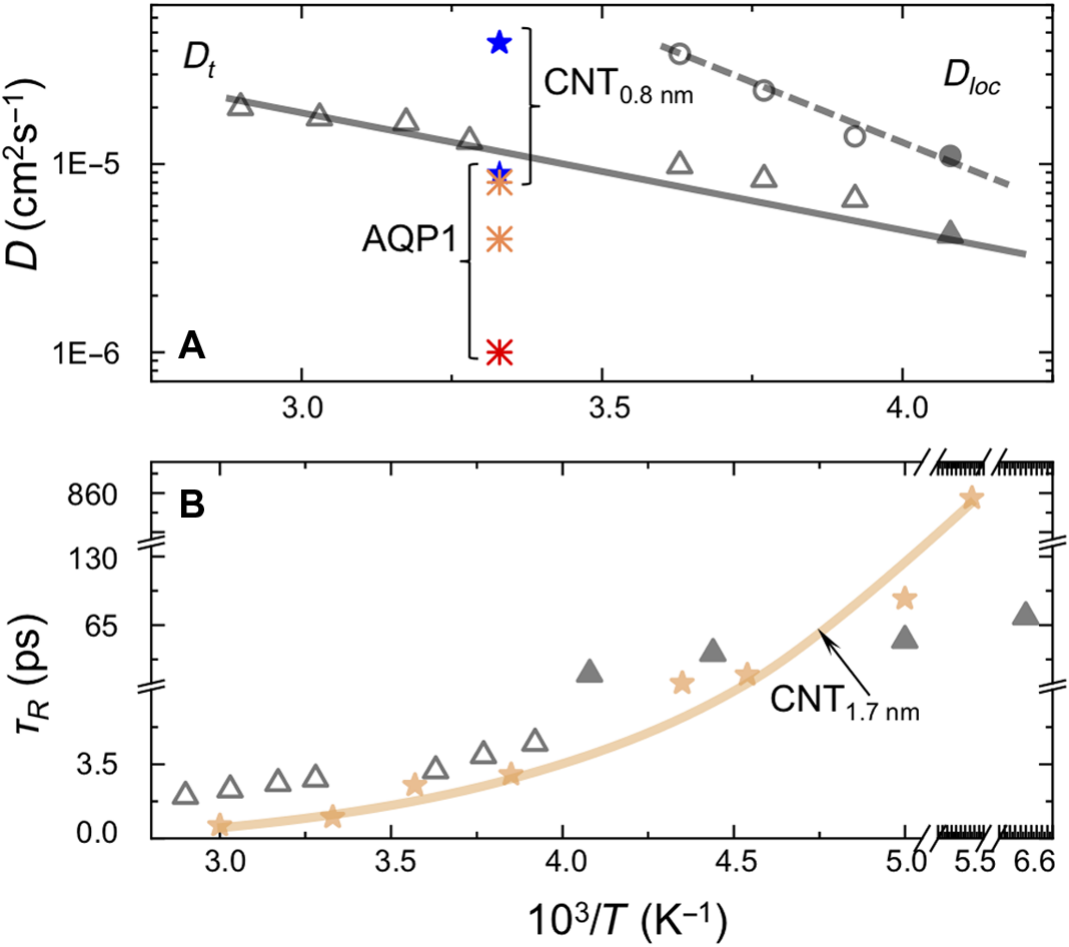
Rapid water transport dynamics in PTI·H_2_O compared with mobility in other nanoconfined environments. (**A**) Translational (*D_t_*) and localized (*D*_loc_) H_2_O dynamics within PTI·H_2_O as a function of temperature (*T*). QENS data were acquired at OSIRIS (open triangles and circles) and IN16B-BATS (filled symbols). *D_t_* data for PTI·H_2_O are compared with measurements for water transport through AQP1 (asterisks) (*14*, *33*) and single-walled CNTs with subnanometer internal diameter (stars) (*14*). (**B**) Relaxation times (τ*_R_ = ħ/*Γ*_R_*) from analysis of the *Q*-independent broad QENS component for PTI·H_2_O between 345 to 150 K (Fig. 3C) compared with water contained within larger-diameter (1.7 nm) CNTs (light brown) (*29*).

Our QENS results revealed a narrow Lorentzian contribution with a dispersive Γ*_T_*(*Q*^2^) relation due to diffusional motion of water molecules in the PTI·H_2_O structure for temperatures between 245 and 345 K (Fig. 3A). Data analysis was carried out using a molecular jump model that provided temperature-dependent values for the translational diffusion coefficient *D_t_*. At 300 K, *D_t_* = 1.32 × 10^−5^ cm^2^/s, faster than values reported for molecular transport through AQP transmembrane channels (Fig. 4A). Our determined diffusion values are comparable with those for H_2_O mobility measured within subnanometer CNTs embedded in lipid vesicles at neutral pH (*14*–*15*), while similar diffusion rates have been determined from studies of water permeability through membranes formed by GO nanosheet assemblies (table S2) (*10*–*11*). The even faster H_2_O transport dynamics reported for subnanometer CNTs, shown in Fig. 4A, were obtained at low pH where protonation of carboxylate groups at the nanotube rim likely plays a key role (*14*–*17*).

Detailed examination of the dispersive Γ*_T_*(*Q*^2^) relations at low *Q* revealed a *Q*-independent region extending up to 0.3 to 1 Å^−1^, depending on the temperature (Fig. 3B). This observation indicates the presence of a nanoconfined regime for H_2_O molecular motions contained within PTI-layered structure. The nanoconfinement radius (*a*) established both from these QENS results and analysis of elastic incoherent structure factor (EISF) data is ~0.5 nm (Fig. 3D), which is comparable with the dimensions of the water mobility “channels” within and between adjacent PTI layers. The QENS analysis provides a value for the localized mobility *D*_loc_ (Fig. 4A) (*21*–*23*). These motions are described in detail by our DFT and ab initio molecular dynamics (AIMD) simulations of water dynamics in PTI·H_2_O in the next section.

Some of the QENS datasets also required fitting a broader Lorentzian component with a *Q*-independent HWHM (Γ*_R_*) (Figs. 2A and 3C). In bulk water and aqueous solutions, such a signal is usually assigned to rotational relaxation dynamics of H_2_O molecules. In the case of water intercalated within the PTI·H_2_O structure, the Γ*_R_* relaxations correspond to dynamics of nanoconfined molecules that are spatially separated from each other and are constrained by H-bonding interactions with the local structural environment. These relaxation processes could be detected down to 150 K with τ*_R_ = ħ/*Γ*_R_* values that are compared with similar dynamics reported for H_2_O in 1.7-nm-diameter CNTs (Fig. 4B and table S2). The QENS data for τ*_R_* agree well with data from nuclear magnetic resonance (NMR) measurements and molecular dynamics simulation results for nanoconfined water in other systems (*11*, *14*, *24*–*29*).

### DFT calculations and AIMD simulations of water mobility mechanisms in PTI·H_2_O

**Fig. 5:**
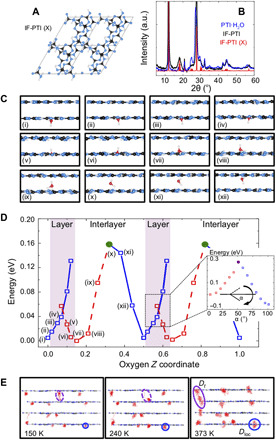
PTI·H_2_O and IF-PTI structures and H_2_O dynamics from DFT and AIMD calculations. (**A**) The equilibrium structure of IF-PTI (configuration X) viewed down the *c* axis. (**B**) Calculated XRD pattern for IF-PTI (X) compared with experimental data for IF-PTI and PTI·H_2_O. (**C**) A series of DFT results showing how an H_2_O molecule initially positioned by OH···N and NH···O interactions relative to the starting PTI layer passes through the intralayer C_12_N_12_H_3_ ring void and into the adjacent layer. In the stable orientation, one OH bond is oriented toward the interlayer space. The H_2_O orientation is reversed by reorganization of the H-bonding interactions. As the molecule traverses the interlayer spacing, it undergoes a further orientation reversal as H bonding to the next layer occurs. The process is then repeated as the molecule traverses further layers and interlayer gallery spacings. (**D**) The energy profile as the H_2_O molecule passes through the configurations labeled (i) through (xii). Blue and red symbols indicate upward- versus downward-pointing orientations. The position of the PTI layers is indicated with a shaded pink area; it is not atomically flat due to some extent of buckling. Configurations generated by maintaining the initial orientation as H_2_O is pushed through the ring become metastable at ~0.05 eV above the ground state. An additional energy cost of ~0.3 eV is required to break the initial H-bonding configuration and reorient the molecule to its more stable configuration (inset showing energy as a function of α as the OH···N angle). (**E**) Oxygen trajectories for H_2_O molecules intercalated in PTI layers from AIMD simulations at three temperatures. Molecules that do not experience the interlayer crossing exhibit dynamics that explore the intralayer C_12_N_12_H_3_ ring voids accompanied by a reversal in molecular orientation (blue circles) (Fig. 4C). Along with the H_2_O dynamics recorded at lower temperature, these nanoconfined motions define the *D*_loc_ dynamics described by QENS analysis.

We first applied DFT calculations to establish the equilibrium geometry of IF-PTI and PTI·H_2_O. The minimum energy configuration for IF-PTI (termed "X" in this study) has a structure in which the C_12_N_12_H_3_ rings are located immediately above each other in successive layers (Fig. 5A). The calculated XRD pattern provides an excellent match with that of the experimentally determined result for IF-PTI (Fig. 5B). This also matches the XRD pattern observed experimentally for PTI·H_2_O, indicating that the structure with superposed intralayer ring pores is maintained as water is included into the structure. The DFT calculations for PTI·H_2_O show that the equilibrium position of water has the oxygen atom located slightly above or below the PTI layer, with one OH bond nearly within the plane of the C_12_N_12_H_3_ void and the other perpendicular to the layer, pointing away from the layer and into the interlayer space (Fig. 1, F and G). Attempts to dissociate water into hydrogen and hydroxide ions always returned neutral H_2_O upon geometry optimization, indicating that the mobility detected by our QENS studies corresponded to diffusion or nanoconfined displacements of intact water molecules within the PTI·H_2_O structure. Our geometry optimizations of the IF-PTI and PTI·H_2_O structures revealed two alternative (X and Z) configurations with slightly displaced and distorted stacking arrangements of the C_12_N_12_H_3_ rings, although both configurations retain the overlap of the void spaces between layers (fig. S2). These had energies that were only 0.001 or 0.007 eV per C_2_N_3_H formula unit higher than the equilibrium structures, depending on the absence or presence of absorbed water. That result is consistent with our AIMD observations discussed below that found that some slight buckling and relative layer displacements occurred as H_2_O molecules transited through the PTI-layered structures. However, the alignment of the intralayer C_12_N_12_H_3_ ring nanopores is retained along the *c* direction independent of the water concentration. Any attempts to displace the H_2_O molecules laterally within the interlayer spacing resulted in rapid energy increases on the order of >1.5 eV per molecule.

To examine the energetics of water transport across the interlayer spacing and through the C_12_N_12_H_3_ rings into the next layer, we conducted series of constrained geometry optimizations (Fig. 5, C and D). Starting with a downward-pointing molecule close to its equilibrium location just below the lower layer [configuration (i) in Fig. 5C], the molecule is translated upward through the C_12_N_12_H_3_ void [configurations (i) to (iv): blue squares in Fig. 5D]. Configurations calculated while maintaining the initial H_2_O orientation showed a rapid increase in energy as the molecules passed through the intralayer pore. A much lower energy configuration was obtained once the H_2_O molecules were permitted to adopt an optimized geometry in which their orientation became reversed in response to the local H-bonding environment. As the molecules traversed the nanopore to emerge into the next layer, the energies of these molecules with reversed orientation decrease toward a minimum as the molecules adopt an "upward" orientation just above the plane of the initial layer (Fig. 5C; red squares in Fig. 5D). As the molecules translate across the interlayer spacing, the energy first increases (red squares) and then begins to decrease (blue squares) as their orientation is reversed a second time due to a reorganization of H-bonding pattern to both the lower and upper layers [Fig. 5, C and D: configurations (viii) through (xii)]. As the H_2_O molecule bonds in its downward-pointing orientation to the upper layer, the ground-state energy of configuration (i) is reestablished, and the process can then be repeated as the molecule passes through the second nanopore into the layer above (Fig. 5D). The crossing between the upward- and downward-pointing curves occurs at an energy ~0.05 eV (~5 kJ/mol) above the equilibrium configuration as the molecule passes through the PTI nanopore (pink region highlighted in Fig. 5D). However, the energy barrier associated with breaking the original set of H bonds formed as the molecule that transits through the nanopore is higher, ~0.3 eV (~30 kJ/mol) (Fig. 5D, inset). This range of energetic barriers is comparable with those determined from temperature-dependent H_2_O mobilities measured in the QENS studies (Fig. 4A). The DFT results show that as the water molecules pass via the C_12_N_12_H_3_ nanopores into the next layer and then traverse the interlayer gallery space, they undergo two sequential reversals in their molecular orientation, as a result of reorganization of the H-bonding patterns between the H_2_O molecules and the ─N═ or ─NH─ species surrounding the C_12_N_12_H_3_ rings within the PTI layers.

We continued our study of water diffusion and nanoconfined mobility within PTI·H_2_O with a series of AIMD simulations carried out at 150, 240, and 373 K for a four-layer model of the structure (Fig. 5E). Our results shown by tracking the oxygen trajectories reveal the emergence of water displacements corresponding to both the *c*-*o*-*m* diffusional processes and nanoconfined dynamics revealed by the QENS analysis. At 150 and 240 K, the AIMD results show that the molecules mainly undergo thermally excited nanoconfined motions about their equilibrium positions with an averaged Debye-Waller radius that increases systematically with temperature. This observation is consistent with our experimental QENS and EISF data analyses described above. A few jumps across the interlayer spacing are also apparent in the AIMD snapshots at these low temperatures, and these also contribute to the *D*_loc_ dynamics observed by QENS (Figs. 3A and 4A). The measured *D*_loc_ values and relaxation times (τ*_R_ = ħ/*Γ*_R_*) show an Arrhenius temperature dependence contrasting with the larger assemblies of supercooled water molecules contained within larger-diameter CNTs and other nanoporous media (table S2). The *c*-*o*-*m* displacement trajectories observed in our AIMD simulations at 373 K correspond to the onset of longer-range diffusional dynamics (*D_t_*) measured in our QENS investigations over length scales up to 40 Å. These diffusional pathways extend across the interlayer spacing and traverse the PTI layers via the C_12_N_12_H_3_ pores according to the configurational energy profile indicated by our DFT calculations (Fig. 5D).

### Water permeance and permeability in PTI membrane structures

Although solution-diffusion models developed for homogeneous media are often applied to rationalize and predict membrane performance, these do not take account of molecular to nanoscale structural heterogeneities. Coronell and co-workers (*30*) have introduced descriptive strategies to account for such “microscale variations” using careful weighting of contributions based on some knowledge of morphology and connectivity. On the basis of this phenomenological model and using our knowledge of the crystalline structure and water self-diffusion coefficients (*D_t_*) within the polycrystalline solid, we estimated the specific permeance (*P* as the permeability, *P_w_*, per unit thickness) for hypothetical PTI membranes using

$$\frac{A}{\nu }=\frac{D_{t}∙K}{\delta }∙\frac{C_{\text{bulk}}\nu }{R∙T}$$ (1)

Here, *A* is the permeability coefficient to be determined with ν the molar volume of water, δ the membrane thickness, *C*_bulk_ is a constant (55 × 10^3^ mol/m^3^) used to model the concentration of bulk water, and *R* and *T* are the ideal gas constant and absolute temperature, respectively. *K* is the water partition coefficient expressed as the molar amount of water included in the membrane as a function of its thickness [(m_w_/(Mw_water_ δ]/C_bulk_, where m_w_ and Mw_water_ are the mass in grams and molecular weight of water) for a hypothetical membrane 10 nm thick containing 1.35 ng of water (table S3). Using our *D_t_* values, we obtained *P* = 400 liter/(m^2^ hour^−1^ bar^−1^) at 300 K (table S3). This prediction is similar to that previously reported for composite CNT/GO membranes (*31*), and it exceeds by several orders of magnitude the averaged values for reverse osmosis membranes (*32*). If we model the performance of an even thinner PTI membrane (e.g., 1-nm thick), then we obtained a value that approaches the permeance of a freestanding graphene layer (table S3). For comparison, we also estimated the water permeability (*P_w_*; in cubic centimeter per second scale) according to the approach suggested by Tunuguntla *et al.* (*14*), where the mobility of single-file water molecules within narrow-diameter CNTs was described by the Einstein relation. Using this alternative approach, we obtained estimates that exceeded by ~30% those reported for subnanometer CNTs at neutral pH (table S3) (*14*).

## DISCUSSION

Recent research in identifying materials for advanced separation science has focused on producing composite membranes based on GO or CNT assemblies (*9*, *10*, *12*, *14*), because of the ultrafast fluxes for water transmission that can be achieved (*31*–*32*). However, attaining these high-performance values requires implementing structural modifications including the employment of grafting species between GO sheets (*8*) or reducing the pH to modify the CNT at its rim to facilitate water entrance (*14*–*17*). Our results show quasi–one-dimensional H_2_O diffusion through the aligned series of stacked C_12_N_12_H_3_ ring nanopores within crystalline carbon nitride with a PTI-layered structure. The diffusion occurs at a rate comparable to those observed in subnanometer CNTs, through GO nanosheet assemblies, and natural AQP channels. The diffusion mechanism involves a concerted sequence of orientation reversals as the molecular H_2_O species traverse the interlayer spacing and pass through the intralayer pores, which resembles the transport mechanism for water molecules through the neck region of AQP channels (*2*–*3*, *33*). The natural transmembrane AQP channels are typically highly selective for transport of molecular H_2_O. That is not only partly due to the narrow diameter of the central channel portion (8 to 10 Å), but is also partly achieved by extracellular gating mechanisms. The C_12_N_12_H_3_ nanopores within the PTI layers are only slightly larger (10 to 12 Å), but we know from structural studies that these can accommodate Cl^−^ ions, but not Br^−^ or larger species, while small cations such as Li^+^ can remain bonded in the place of N─H hydrogens surrounding the pore interior. We do not yet know the relative diffusivity or permeance of these other species within and through the PTI-layered structures, so it is not yet possible to estimate the ionic versus molecular selectivity of such PTI membranes. However, the ultrafast water diffusivity and permeance occurring at rates similar to those observed in subnanometer CNT and GO assemblies, and that exceed those observed in natural AQP channels, mean that these PTI-layered nanoporous materials represent excellent candidates for inclusion in membrane systems for next-generation energy and nanofiltration device applications.

## MATERIALS AND METHODS

### Sample preparation

The starting PTI·LiCl crystals used in this study were obtained by polymerization of dicyandiamide (C_2_N_4_H_4_) in a LiCl/KCl eutectic molten salt mixture in an evacuated SiO_2_ glass tube at 600°C. After synthesis, excess salt was removed by washing in water and ethanol. The resulting intercalated crystalline solids were determined to have composition C_12_N_17.5_H_6.3_Cl_1.5_Li_3.2_ using a combination of x-ray photoelectron spectroscopy (XPS), bulk analysis, and solid-state ^7^Li NMR techniques (*34*). The crystalline materials were characterized using total powder scattering with pair distribution function (PDF) analysis using high-energy x-rays at the Advanced Photon Source beamline 6IDD (incident energy of 60.04 keV and λ = 0.2064 Å) and laboratory powder XRD (CuK_α_ radiation). The intercalated Li^+^ and halide ions were removed by continuously washing the samples in H_2_O via Sohxlet extraction over a period of 15 to 21 days. The result was a fully deintercalated crystalline PTI material (IF-PTI) with a layered structure and composition C_6_N_9_H_3_ (*19*). The reversible uptake of atmospheric H_2_O was determined by TGA and FTIR spectroscopy with changes in layer stacking and interlayer spacings studied by powder XRD. TGA was performed using a Perkin Elmer Pyris 1, with ~2 mg of material heated from 100° to 900°C at 10°C min^−1^ under a 60 standard cubic centimeter per minute flow of compressed air, recorded at 0.2-s intervals. Samples for neutron studies were loaded into thin-walled annular Al cells and sealed under different conditions: (i) H_2_O-free conditions within an Ar atmosphere environment to study IF-PTI samples, (ii) sealed inside the cells following exposure to ambient air to study fully intercalated PTI·H_2_O, or (iii) placed inside the cans, along with a determined amount of liquid water, to study fully saturated PTI·H_2_O in the presence of excess water, or PTI samples with H_2_O molecules adsorbed on external surfaces.

### Neutron scattering

#### Dynamical contributions to the neutron scattering of nanoconfined H_2_O

QENS analyzes the broadening that appears around the elastic scattering signal due to dynamical relaxation processes. Because of their large incoherent neutron cross section, the scattering is dominated by the dynamical response of protons within the sample. The central signal is modeled by an instrumental resolution profile convoluted with a delta function [δ(ω)] that includes any protons that appear “immobile” within the spectroscopic energy-transfer window (Δ*E* in microelectronvolt) probed by the neutron scattering experiment. Proton dynamics occurring much faster than the energy-transfer window lead to an approximately flat background in the observed datasets (Fig. 2B). The QENS broadening was analyzed by examining the correlation of the energy-transfer linewidth (Γ, defined as the HWHM) that determines the relaxation time (τ) for the process involved as a function of the momentum transfer (*Q*). It is further possible to discriminate between (i) fully diffusional motions that result in a dispersive Γ(*Q*^2^) relation (*Q*-dependent; purple line in Fig. 2) and (ii) localized rotational-translational or librational movements, which provide a nondispersive Γ(*Q*^2^) relation (*Q*-independent; green line in Fig. 2) (*21*). In studies of bulk water, this second component that gives rise to the broader Lorentzian contribution is interpreted to represent a convolution of translational displacements with additional motions, including rotational relaxation or reorientation of the H_2_O molecules (*21*, *35*).

The diffusional QENS that results from *c*-*o*-*m* displacements leads to a dispersive Γ(*Q*^2^) relation (*Q*-dependent) from which a translational diffusion coefficient (*D_t_*) can be extracted using a jump diffusion model, assuming that the molecules execute oscillations during a mean residence time (τ_0_) then jump during a shorter time τ_1_ to a second site with τ_0_ » τ_1_ (*21*, *35*)

$$S_{T}(Q,\omega )=\frac{1}{\pi }\frac{\Gamma_{T}}{\omega^{2}+{\left(\Gamma_{T}\right)}^{2}}$$ (2)

$$\Gamma_{T}=\frac{D_{t}Q^{2}}{1+Q^{2}D_{t}\tau_{0}}$$ (3)

Under spatial confinement, water is generally found to exhibit longer residence times, as expected from percolation-type models and a departure from the bulk structure of water, and new types of diffusive modes emerge. Confinement is manifested in two main features appearing in the QENS data (*22*–*23*): (i) the emergence of an elastic component in the dynamic structure factor (EISF) and (ii) a linewidth Γ that becomes practically invariant at low *Q*, leading to

$$\Gamma_{0}=4.33\frac{D_{\text{loc}}}{a^{2}}$$ (4)

We thus rewrite Eq. 2

$$S_{T}(Q,\omega )=A_{0}(Q,T)\delta (\omega )+(1-A_{0}(Q,T))\frac{1}{\pi }\frac{\Gamma_{T}}{\omega^{2}+{\left(\Gamma_{T}\right)}^{2}}$$ (5)

Here, *A*_0_(*Q,T*) is the EISF and δ(ω) is the delta function representing the elastic peak, leading to

$$A_{0}(Q,T)=\text{EISF}(Q)=p+(1-p){\left\{\frac{3[\text{sin}(\mathrm{Qa})-(\mathrm{Qa})\text{cos}(\mathrm{Qa})]}{{\left(\mathrm{Qa}\right)}^{3}}\right\}}^{2}$$ (6)

where *p* is the fraction of immobile protons in the system under investigation.

#### Neutron scattering instruments and facilities

Our incoherent QENS experiments were carried out using instruments at different neutron scattering facilities to take advantage of the different spectrometer resolutions available. Initial studies were performed at the OSIRIS time-of-flight (TOF) near-backscattering indirect spectrometer at the ISIS facility, UK. The PG002 configuration was used to examine energy transfers over a *Q* range 0.27 to 1.80 Å^−1^, with energy resolution (ΔE) of 25 μeV and a dynamic range of either ±0.6 meV or −0.5/+1 meV. We applied an offset only in the case of hydrated PTI sample to investigate its water relaxation dynamics. To expand the dynamical range and better characterize the water dynamics of our system, we ran further experiments at higher-resolution instruments at Institut Laue-Langevin (ILL; Grenoble, France) and National Institute of Standards and Technology (NIST; USA). We used IN16B at ILL in BATS (Backscattering And Time-of-Flight Spectrometer) configuration (i.e., inverted TOF on back-scattering spectrometers) to examine energy transfers over a *Q* range 0.29 to 1.84 Å^−1^, with energy resolution (Δ*E*) of 3.5 μeV and a dynamic range of −0.15/+0.17 meV (*41*). We also used the High Flux Backscattering Spectrometer (HFBS) at NIST to examine energy transfers over a *Q* range of 0.25 to 1.75 Å^−1^, with energy resolution (Δ*E*) of 1 μeV and a dynamic range of ±15 μeV (*42*). This combination of experimental conditions allowed us to investigate the mechanisms, geometry, and frequency of motions over a broad time window, ranging from tens of picosecond to nanosecond.

#### QENS data analysis

The scattering function [*S*(*Q,*ω)] that represents the time Fourier transform of the intermediate scattering function [*I*(*Q,t*)] contains information about the spatiotemporal correlation between identical nuclei (*S*_inc_) and the static and dynamic correlations of distinct nuclei (*S*_coh_) (*21*). *S*_inc_ is typically decoupled into translational (diffusional) and hindered rotational/reorientational contributions, assuming that these motions are independent and are modeled by Lorentzian functions. All immobile protons (i.e., those moving slower than the instrumental resolution), along with coherent structural components, give rise to an elastic scattering term [δ(ω)], while any faster dynamics are incorporated in the background that appears flat and featureless in the QENS spectral analysis. δ(ω) convoluted with the instrumental resolution (determined using a vanadium standard) could be accounted for by using the scattering profile at 5 K. Our raw data were normalized to the neutron flux and detector efficiency fluctuations by direct comparison with the purely incoherent signal from a vanadium reference. The double differential cross section was then converted into the corresponding dynamic structure factor *S*(*Q*,ω). The detectors were grouped to provide around 11 to 16 spectra and the energy binned into constant 0.0005 or 0.005 meV steps. Additional normalization steps, such as removing the signal associated to the empty can, and data analysis were carried out directly on the *S*(*Q*,ω) spectra using Mantid (*36*), DAVE (*37*), or Origin 2019b. Each *Q* slice was analyzed using a built-in least squares algorithm, accounting for the instrumental energy resolution along with up to two Lorentzian functions.

#### DFT and AIMD calculations

DFT calculations to study the locations and bonding of H_2_O molecules within the interlayer sites and to predict energy barriers for transport within and between the layers were carried out using the Perdew–Burke-Ernzerhof (PBE) functional with D3 corrections for the dispersion forces as implemented in the code CRYSTAL17 (*38*). All-electron Gaussian basis sets available from the CRYSTAL online database were used, indicated by the following labels: C (C_6-31d1G_gatti_1994), N (N_6-31d1G_gatti_1994), H (H_3-1p1G_gatti_1994), and O (O_6-31d1_gatti_1994). A Monkhorst-Pack shrinking factor of 2 × 2 was used, leading to 8 *k* points in the irreducible Brillouin zone. The threshold parameters for the Coulomb and exchange series were set at default values 7, 7, 7, and 14, while the self-consistent field (SCF) calculation was performed up to the convergence threshold at 1 × 10^−7^ Hartree. Migration barriers were calculated in CRYSTAL using a sequence of constrained geometry optimizations. For the intravoid site migration, the distance between water oxygen and the imide bridging nitrogen toward which the water molecule is migrating was constrained with a step size of ~0.07 Å. For the intrasheet crossing migration, the angle between the imide bridging nitrogen, water oxygen, and the water hydrogen pointing out of the plane of the PTI C_12_N_12_H_3_ ring is constrained, and sequential partial geometry optimizations with a step of ~5° were performed to capture in important rotational dynamics of the system. For the interlayer translation, the *z* coordinate of the migrating water oxygen and an arbitrary PTI C atom in the same C_12_N_12_H_3_ were constrained and varied using a step size of 0.1 to 0.5 Å. AIMD calculations were performed using the CP2K package, with implemented BLYP (Becke, Lee, Yang, Parr) functional and GTH-DZVP MOLOPT basis sets and SCF convergence tolerance set to 1 × 10^−7^ Hartree and MGRID/REL CUTOFF cutoffs of 60 and 500 rydbergs, respectively (*39*). Calculations were run at three different temperatures (150, 240, and 373 K) using the CSVR thermostat, with a time step of 0.5 fs for a total of 14 ps.

## Supplementary Material

abb6011_SM.pdf

## SUPPLEMENTARY MATERIALS

Supplementary material for this article is available at http://advances.sciencemag.org/cgi/content/full/6/39/eabb6011/DC1
