# Supplementary material for: Aquaporin-like water transport in nanoporous crystalline layered carbon nitride
Source: Sci Adv. 2020 Sep 25;6(39):eabb6011. doi: 10.1126/sciadv.abb6011 (PMC7518864; doi:10.1126/sciadv.abb6011)
Supplement: abb6011_SM.pdf [file abb6011_SM.pdf]

Science Advances
AAAS

advances.sciencemag.org/cgi/content/full/6/39/eabb6011/DC1

Supplementary Materials for

Aquaporin-like water transport in nanoporous crystalline layered carbon nitride

Fabrizia Foglia, Adam J. Clancy, Jasper Berry-Gair, Karolina Lisowska, Martin C. Wilding, Theo M. Suter, Thomas S. Miller, Keenan Smith, Franz Demmel, Markus Appel, Victoria García Sakai, Andrea Sella, Christopher A. Howard, Madhusudan Tyagi, Furio Corà, Paul F. McMillan*

*Corresponding author. Email: p.f.mcmillan@ucl.ac.uk

Published 25 September 2020, *Sci. Adv.* **6**, eabb6011 (2020)
DOI: 10.1126/sciadv.abb6011

This PDF file includes:

Supplementary Text
Figs. S1 to S3
Tables S1 and S2
References

1. Neutron scattering: QENS interpretation and dataset analysis

QENS analyzes the broadening measured as an energy transfer *($E = \hbar\omega$)* function that appears around the base of the elastic scattering signal, resulting from dynamical relaxation processes in the system occurring on a timescale which is compatible with the spectrometer energy resolution window. The elastic signal is modelled using the instrumental resolution function obtained using a vanadium standard convoluted with a $\delta(\omega)$ term that accounts for all dynamics occurring slower than the instrumental resolution. Any faster dynamics are incorporated in the background that appears flat in the observed spectrum (Figures 2A,B; Figure S1). The scattering function ($S(Q,\omega)$) is composed of coherent and incoherent terms reflecting the spatio-temporal correlations among systems of identical nuclei ($S_{inc}(Q,\omega)$) and those of distinct nuclei (S_{coh}), as a function of the momentum (Q) and energy exchange (*21*).

Considering the incoherent scattering term, the measured QENS signal can be deconvoluted into vibrational (S_V), rotational (S_R) and translational (S_T) components :

$$S_{inc}(Q,\omega) = S_V(Q,\omega) \otimes S_R(Q,\omega) \otimes S_T(Q,\omega)$$
(S1)

In the case of isotropic, harmonic vibrations, the vibrational contribution can be expressed in terms of the mean-square displacement ($\langle u \rangle$), and Eq. S1 is rewritten as:

$$S_{inc}(Q,\omega) = e^{-Q^2\langle u^2\rangle/3} \otimes S_R(Q,\omega) \otimes S_T(Q,\omega)$$
(S2)

The QENS broadening due to S_R and S_T components is modelled using one or more Lorentzian functions as demanded by the individual datasets or the dynamical relaxation model being considered (Figures 2A,B; Figure S1). The resulting Lorentzian fits are analyzed by examining the correlation of the linewidth (Γ, HWHM) that determines the relaxation time (τ) for the process involved as a function of Q. Following this procedure we can discriminate between: i) diffusional processes that involve *c-o-m* displacement and ii) localized rotational-translational movements.

Diffusional motions result in a dispersive $\Gamma(Q^2)$ relation. In the case of bulk and nanoconfined water, such relations are analyzed according to one of a hierarchy of possible jump-diffusion models to extract the translational diffusion coefficient (D_t) (*21-23*). The simplest jump-diffusion model applied here appears as Eqs. 2 and 3 in the main paper. Localized movements that do not involve *c-o-m* translation such as pseudorotational dynamics in bulk water and that also arise when water molecules are present in a nanoconfined environment lead to a non-dispersive $\Gamma(Q^2)$ relation that is interpreted in terms of the relaxation time (τ_R). In this case two main features are manifested in the QENS data *(21-23)*: i) the emergence of an elastic component in the dynamic structure factor (EISF) and ii) a linewidth Γ which becomes practically invariant at low-Q. From this latter it possible to extract the local diffusion coefficient (D_{loc}; Eq. 4 main paper) within the confinement region (approximated by a sphere of radius a as shown in Figure 2F of the main text).

2. Neutron scattering experiments and instrument facilities

Samples for neutron studies were prepared by loading ~ 0.9 g of material into thin-walled annular aluminum cells and sealed either i) in ambient air in order to study PTI·H_2O as a function of temperature (Figures 2A-F) or ii) in a dry (<1 ppm H_2O) Ar-

filled glovebox to examine the dynamics of IF-PTI (Figure S1). Additional experiments were carried out for PTI samples intentionally hydrated with excess water (up to 30% by weight) (see below and Figure S3). Data were acquired between 5 and 345 K. QENS profiles were measured using a combination of time-of-flight near back-scattering ($\Delta E = 25$ μeV; OSIRIS, ISIS, UK) and high-resolution backscattering ($\Delta E = 3.5$ μeV; IN16B-BATS, ILL, France and $\Delta E = 1$ μeV; HFBS, NIST, USA) spectrometers. This range of instruments allowed the examination of nanoconfined and translational diffusive water motions over a broad time window spanning from tens of picoseconds to several nanoseconds. The results of our data analyses are summarised in Table S2 along with comparisons with D_T, D_{loc} and τ_R values for materials examined in previous experimental and simulation studies.

We began our investigation by testing, IF-PTI and PTI·H_2O (containing ~9 wt% water). The analysis performed over a broad time window (tens of ps to ns) confirmed that QENS broadening was only visible in the case of PTI·H_2O, and therefore only related to the water movement (Figures 2A,B, Figure S1). In the tens of ps time scale ($\Delta E = 25\ \mu eV$) the data indicate that diffusive dynamics enter in the spectroscopic window above 250 K. Extending the investigation to ns ($\Delta E = 3.5\ \mu eV$) timescales it was clear that localized motions were visible from 150 K whereas diffusive dynamics entered the spectroscopic window just above 240 K. These dynamics were significantly slowed with respect to bulk water, but were comparable with water confined in different nanoporous matrices including Vycor glasses, Nafion, CNT and GO (Table S1).

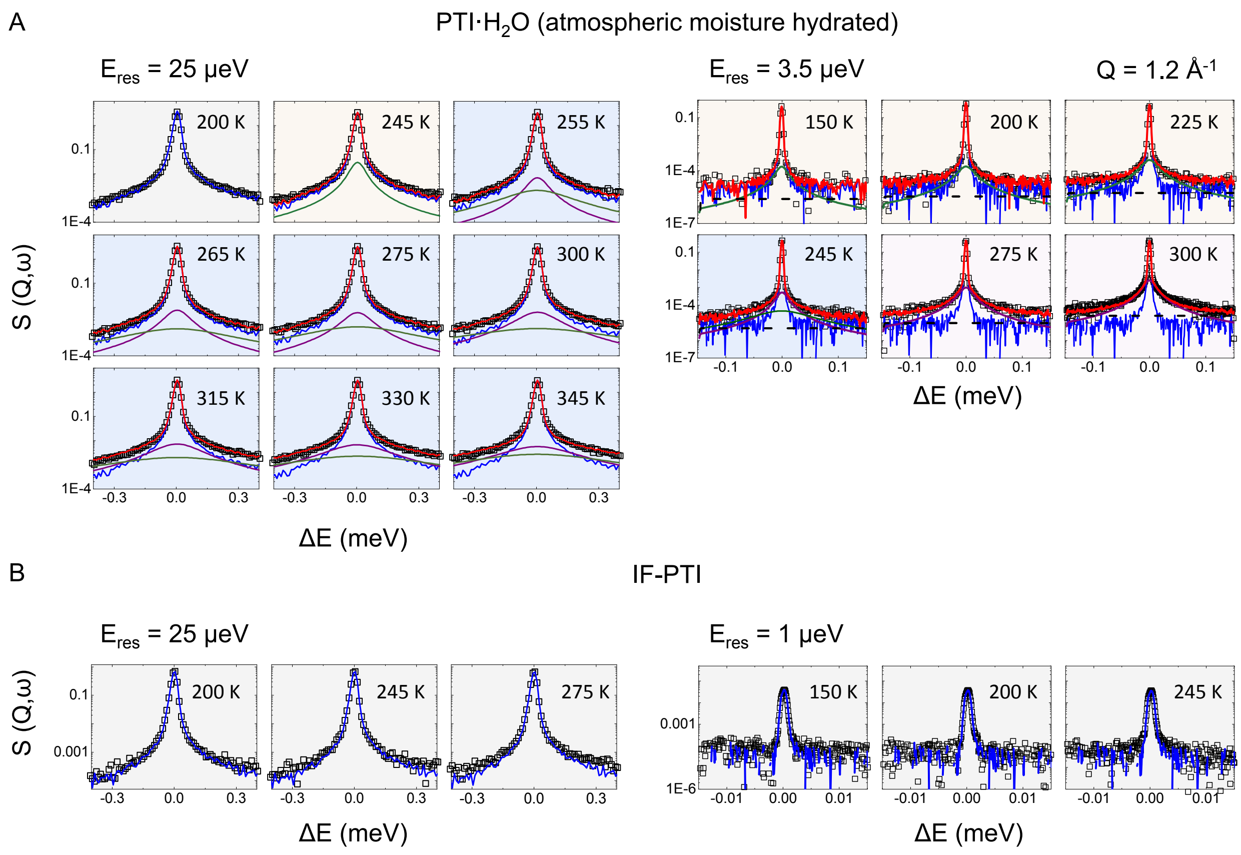

Figure S1. QENS data obtained for PTI before (IF-PTI) and after exposure to atmospheric moisture (PTI·H_2O containing ~9 wt% H_2O intercalated within the structure). Experiments have been performed at three spectrometers with different instrumental resolutions: E_{res} = 25 μeV (OSIRIS, ISIS, UK) (*40*), 3.5 μeV (IN16B, ILL, Grenoble) (*41*) and 1 μeV (HFBS, NIST, US) (*42*) US) to investigate a time scale ranging from ns to a few tens of ps. Raw data and lineshape fits are shown for Q = 1.2 $Å^{-1}$ with a logarithmic intensity scale to highlight QENS contributions. The global fit (red continuous curve) is overlain on the ata points (black). The instrumental resolution profile convoluted with the *δ (ω)* function obtained at 5 K is shown in blue. (**A**) Lorentzian components are present in the QENS profile by 150 K for PTI·H_2O (top panels). These represent a convolution of localised relaxational (rotational-translational or librational, green) and/or *c-o-m* (i.e., translational diffusion, magenta) contributions. (**B**) In contrast, no QENS signal was observed for IF-PTI between 5 to 275 K.

Table S1. Comparison of dynamical data for water mobility in bulk and nanoconfined within different matrices. Translational (D_t), nanoconfined (D_{loc}) and rotational (τ_R) relaxation dynamics for translationally mobile H_2O molecules in different environments. The comparison was made over a wide range of temperatures and conditions. Data for PTI·H_2O were acquired at two spectrometers with energy resolutions E_{res} = 3.5 and 25 μeV and analyzed using Eqns. 3 and 4. The dynamics for PTI·H_2O are significantly different to those observed for stable (*43-44*) and supercooled liquid water (*45*), although the differences become less for H_2O samples nanoconfined within Vycor® or partially hydrated Nafion® matrices (*46*). We also compared our results for PTI·H_2O with data obtained for water mobility in AQP1 (*14,33*), CNT (*14,25-29*) and intercalated within GO (*10-11*).

**T (K)**	**Sample**	**Water uptake**	**Technique**	**D_t 10^{-5} (cm^2/s)**	**D_{loc} 10^{-5} (cm^2/s)**	**τ_R (ps)**
**150**	PTI	9 wt%	$QENS_{3.5\ \mu eV}$	-	-	73.0
**180**	CNT (2a = 1.7 nm)[29]	20-57 wt%	NMR / MD	-	-	~800
**200**	CNT (2a = 1.4 nm)[26]	11 wt%	$QENS_{1.2\ \mu eV}$	0.16	-	-
	PTI	9 wt%	$QENS_{3.5\ \mu eV}$	-	-	50.6
**225**	PTI	9 wt%	$QENS_{3.5\ \mu eV}$	-		38.5
**230**	CNT (2a = 1.4 nm)[26]	11 wt%	$QENS_{1.2\ \mu eV}$	0.24	-	-
**235**	Bulk water[45]	-	PLH	0.085	-	-
**240**	CNT (2a = 1.7 nm)[29]	20-57 wt%	NMR / MD			~7.0
	CNT (2a = 1.4 nm)[26]	11 wt%	$QENS_{1.2\ \mu eV}$	0.27	-	-
	Bulk water[45]	-	PLH	0.15	-	-
**245**	PTI	9 wt%	$QENS_{3.5\ \mu eV}$	0.42	1.1	18.8
			$QENS_{25\ \mu eV}$	-	-	28.6
		~30 wt%		-	-	26.0
**250**	CNT (2a = 1.4 nm)[26]	11 wt%	$QENS_{1.2\ \mu eV}$	0.46	-	-
	CNT (2a = 1.4 nm)[25]		$QENS_{70\ \mu eV}$	0.06	-	1.25
**255**	PTI	9 wt%	$QENS_{25\ \mu eV}$	0.65	1.20	4.5
		~30 wt%		0.70	1.55	6.6
**258**	Vycor[23]	100% RH	QENS	0.75	1.40	-
		25% RH		1.20	0.30	-
**260**	CNT (2a = 1.7 nm)[29]	20-57 wt%	NMR / MD	-	-	~3.0
	CNT (2a = 1.4 nm)[26]	11 wt%	$QENS_{1.2\ \mu eV}$	0.54	-	-
**265**	PTI	9 wt%	$QENS_{25\ \mu eV}$	0.83	2.45	3.9
		~30 wt%		0.92	2.30	4.4
**275**	PTI	9 wt%	$QENS_{3.5\ \mu eV}$	0.50	-	7.3
			$QENS_{25\ \mu eV}$	0.98	3.85	3.2
		~30 wt%		1.39	-	2.6
**278**	Vycor[23]	100% RH	QENS	1.40	3.30	-
		52% RH		1.82	1.40	
	Bulk water[43]	-	QENS	1.35	-	1.33
**280**	CNT (2a = 1.4 nm)[29]	20-57 wt%	NMR / MD	-	-	~1.8
	CNT (2a = 1.4 nm)[25]	11 wt%	$QENS_{70\ \mu eV}$	0.36	-	0.84
**285**	PTI	~30 wt%	$QENS_{25\ \mu eV}$	1.75	-	2.0
**293**	Bulk water[43]	-	QENS	2.40	-	1.0
**300**	CNT (2a = 1.4 nm)[29]	20-57 wt%	NMR / MD	-	-	~1.0
	CNT (2a = 1.1 nm)[29]			-	-	~4.0
	CNT (2a = 1.4 nm)[25]	11 wt%	$QENS_{70\ \mu eV}$	1.0	-	0.61
	CNT (2a = 0.8 nm)[27]	-	MD	2.5 (D_z = 4.3; D_{x-y} = 1.6)	-	-
	CNT (2a = 1.1 nm)[27]	-		3.2 (D_z = 3.5; D_{x-y} = 3.0)		
	CNT (2a = 0.8 nm)[28]	-		D_z = 1.16		
	GO (2a = 0.65 nm)[11]	N_w = 58	MD	30.87	-	-
	GO (2a = 0.65 nm)[11]	N_w = 986		1.49	-	-
	GO (2a = 0.6 nm)[10]	-	MD	~0.70		-
	CNT_{porin} (2a = 0.8 nm)[14]	-	MD	11.3 (pH 3.0)	-	-
			Stopped-flow	4.4 (pH 3.0); 0.9 (pH 7.8)	-	-
	AQP1[14]	-	Stopped-flow	0.15	-	-
	AQP1[33]	-	-	0.4 – 0.8	-	-
	Nafion[46]	λ = 3.3	QENS	0.45	-	-
		λ = 17.5		2.0	-	-
**305**	PTI	9 wt%	$QENS_{3.5\ \mu eV}$	0.77	-	-
			$QENS_{25\ \mu eV}$	1.32	-	2.8
		~30 wt%		2.73	-	1.6
**315**	PTI	9 wt%	$QENS_{25\ \mu eV}$	1.66	-	2.6
		~30 wt%		3.60	-	1.3
**330**	PTI	9 wt%	$QENS_{3.5\ \mu eV}$	1.14	-	-
			$QENS_{25\ \mu eV}$	1.76	-	2.3
		~30 wt%		4.30	-	1.2
	CNT (2a = 1.1 – 1.7 nm)[29]	20-57 wt%	NMR / MD	-	-	~0.6
	Bulk water[44]	-	NMR	4.80	-	-
**345**	PTI	9 wt%	$QENS_{25\ \mu eV}$	2.00	-	2.0
		~30 wt%		5.62	-	-

3. DFT calculations and geometry optimization

DFT calculations were employed to study the locations and bonding of H_2O molecules within the interlayer sites, the equilibrium geometry of IF-PTI and PTI·H_2O and to predict energy barriers for transport within and between the layers. Calculations were carried out using the PBE functional with D3 corrections for the dispersion forces as implemented in the code CRYSTAL17 (*38*). The geometry optimized results for IF-PTI revealed two slightly different minimum energy solutions for the stacked layers (labelled X and Z) that differed by less that 0.001 eV per C_2N_3H formula unit (Figure S3A,B). Both resembled the AA' stacking motif of the original PTI·LiCl or PTI·LiBr compounds containing intercalated Li+ and Cl^-/Br^- ions in which the $C_{12}N_{12}H_3$ intralayer voids are aligned along the *c* axis resulting in continuous nanopores for H_2O molecules to travel through the structure. The two structures are related by slight displacements of adjacent layers along the (010) and (210) directions.The IF-PTI (X) configuration provided an excellent match to the experimental XRD pattern for both IF-PTI and PTI·H_2O (Figure 4B in main text). The same two configurations were obtained following geometry optimization of the PTI·H_2O structure (Figure S3C,D) but in this case the X configuration was 0.007 eV per C_2N_3H formula unit lower in energy.

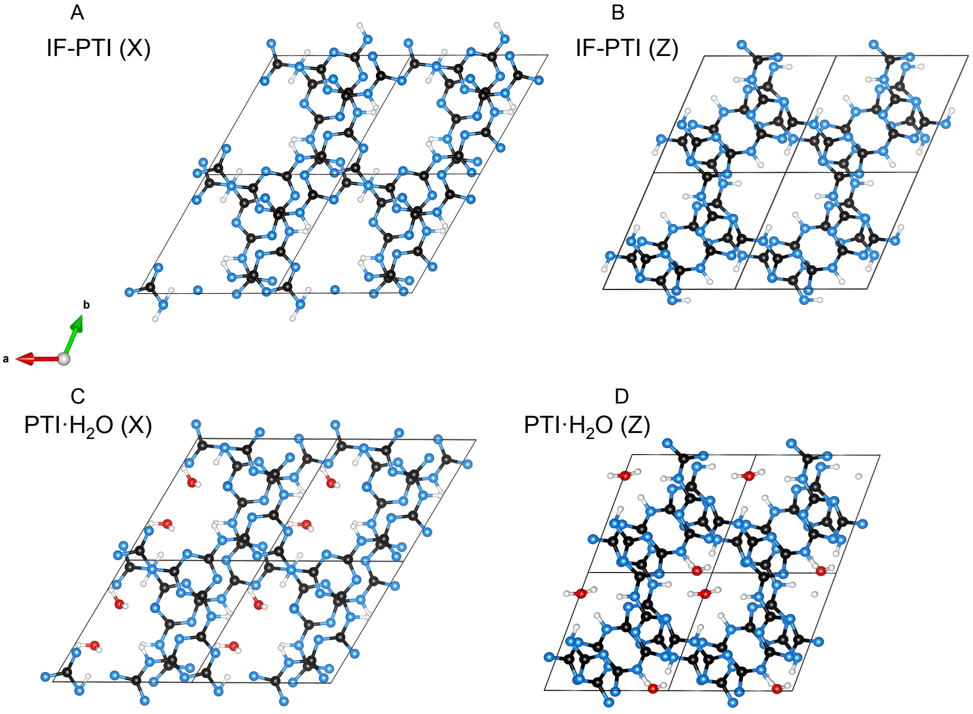

Figure S2. Two different geometry optimized structures, labelled X and Z, for IF-PTI and PTI·H_2O structures, obtained from DFT calculations. Both structures contain overlapping void spaces between successive layers, a feature that enables facile water diffusion along the interlayer channels. Structures X and Z have been obtained starting from an AA' stacking of the PTI layers in space group P6_3 cm, which is the stable phase of PTI·LiCl (*19*), and removing the LiCl intercalants. They correspond to slight displacements of adjacent layers along the (010) and (210) directions respectively. The two structures are nearly isoenergetic, with a difference of less than 0.001 eV per C_2N_3H formula unit, for the IF-PTI material. In presence of water, structure X is stabilised by 0.007 eV per formula unit

over Z, corresponding to 0.021 eV per intercalated water molecule. The alignment of the intralayer $C_{12}N_{12}H_3$ ring nanopores along the *c* direction is therefore retained, independently from the water concentration.

4. Membrane performance

Although solution-diffusion type models are often applied to rationalize engineering performance of membranes (*47*), they do not explicitly account for molecular and nanoscale heterogeneity (e.g. pore morphology and topology). Coronell and co-workers have introduced descriptive strategies to account for these "microscale variations" employing "carefully weighting" of contributions based on some knowledge of morphology and connectivity (*30, 48-49*). Here we implemented this type of model using water self-diffusivity calculated via QENS (Eq. 1 main paper) to predict membrane permeance of hypothetical PTI-membranes having different thicknesses (Table S2). For comparison we also estimated the water permeability (P_w) according to the approach suggested by Tunuguntla et al. (*14*), where the mobility of single-file water molecules was described by the Einstein relation:

$$D = \frac{z^2 P_w}{2 v_w} \quad \text{(S3)}$$

were z is the average distance between two water molecules in the single-file water wire (~2.78 Å), and v_w is the molecular volume of one water molecule. We predicted permeance and permeability values for PTI membranes. Our data for a 1 nm thick membrane predict a permeance of 96 L/(cm^2 day MPa); this figure at 300 K exceeds by several orders of magnitude the averaged value for RO membrane (2.6 10^{-2} L/(cm^2 day MPa)), but is comparable to freestanding graphene single-layer (129 L/(cm^2 day MPa) (Table S2).

Table S2. Water permeance and permeability for different model membranes. Comparison between permeance for PTI membranes with different specifications, modeled using the self-diffusion for bulk PTI. Water permeance (P) is modeled according to Eq. 1 (*30, 48-49*); water permeability (P_w) is modeled according to Eq. S3 (*14*). Comparison is made between a few composite, CNT and GO membranes (*14, 31, 32,50-51*) as well as AQP1 (*52*).

Sample	Membrane characteristics	P (L m^{-2} h^{-1} bar^{-1})	P (L cm^{-2} day^{-1} MPa^{1})	P_w (cm^3 s^{-1})
PTI	1 µm / 135 ng water	4.0	9.6 10^{-2}	-
	10 nm / 1.35 ng water	400.0	9.6	-
	1 nm / 0.135 ng water	4000.0	96.0	-
	-	-	-	1.00 10^{-12}
RO membrane[32]	-	-	~2.6 10^{-2}	-
Functionalised CNT[32]	-	-	10.2	-
CNT/GO composite[31]	-	~30 - ~700	-	-
GO membrane[50]	3 nm	1370	(33)	-
Freestanding GO[51]	-	-	129	-
$CNT_{1.35nm}$[14]	pH 7.8	-	-	0.06 10^{-12}
	pH 3.0	-	-	0.22 10^{-12}
$CNT_{0.68nm}$[14]	pH 7.8	-	-	0.68 10^{-12}
	pH 3.0	-	-	3.40 10^{-12}
AQP1[52]	-	-	-	0.12 10^{-12}

5. PTI treated with an excess of water (~ 30 wt%)

For completeness we also studied PTI·H_2O treated with an excess of water (~30 wt%; see Figure S2, Table S2). The analysis clearly indicates that above the threshold of ~9 wt%, which represents the maximum amount of H_2O that can be intercalated within the structure, any excess is observed to behave as bulk water. Interestingly the dynamics of the highly confined water remain visible in QENS data taken below 265 K; this suggest the presence of two populations of water: i) the intercalated (nanoconfined) H_2O molecules, and excess (bulk) H_2O that is being examined in a deeply supercooled regime. This bulk water signature must be derived from water that is closely associated with the surface of the PTI·H_2O crystals.

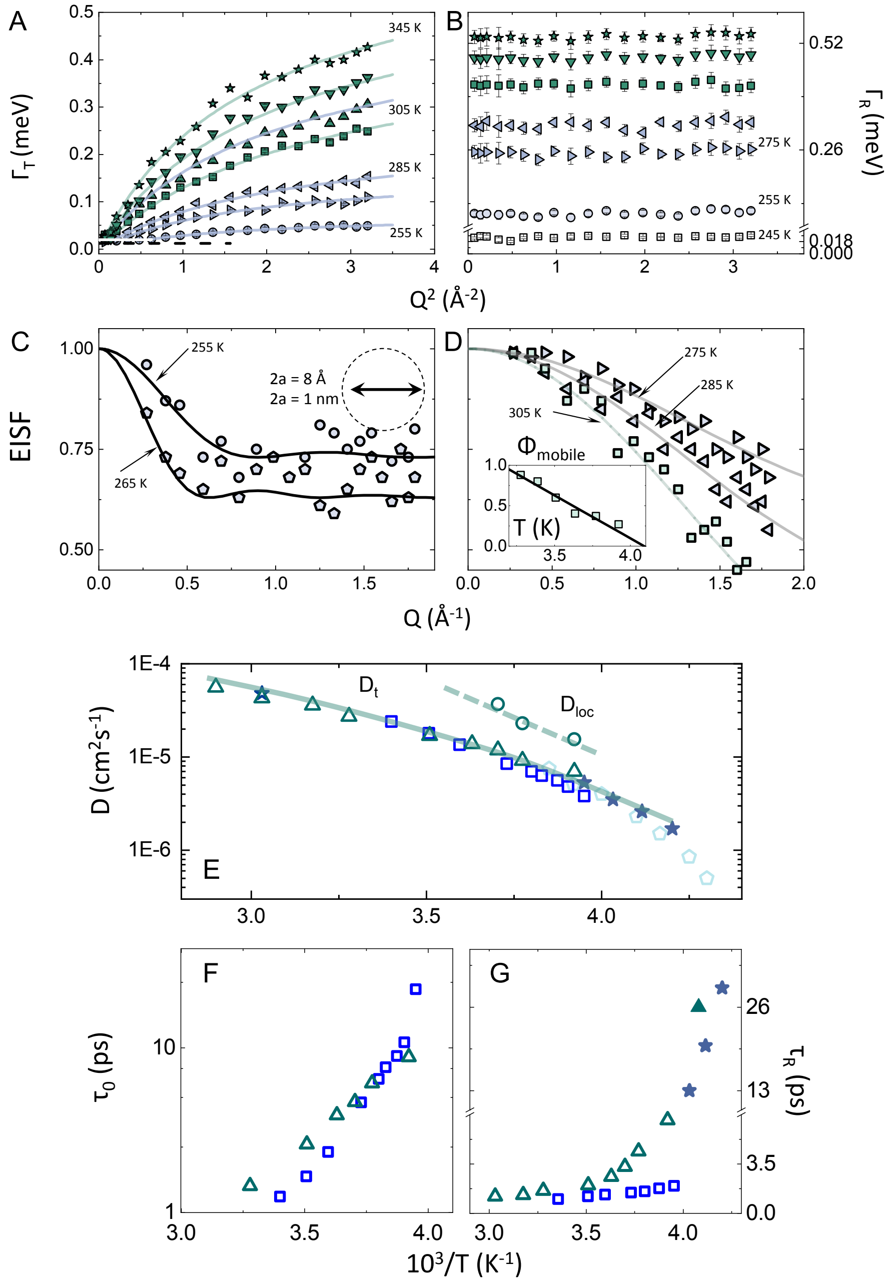

Figure S3. Analysis of QENS and EISF data for PTI·H_2O containing excess (~30 wt%) water included with the sample. Data were obtained at OSIRIS (ISIS, UK) with an instrumental resolution of 25 μeV. (**A**) Dispersive $\Gamma_T(Q^2)$ plot for the narrow Lorentzian component observed between 255 to 345 K indicating *c-o-m* diffusional displacements of the

H_2O molecules. (**B**) Dispersionless $\Gamma_R(Q^2)$ dynamics where Γ_R is: i) the linewidth of the single Lorentzian function found for the system dynamics at low T (255 K), or ii) by deconvolution from the broad (Γ_T + Γ_R) component when two Lorentzian functions were needed to describe the dynamics (255 to 345 K). (**C**) EISF data obtained at OSIRIS. The data could be modelled by fitting to rotation of intercalated and nanoconfined water molecules within a spherical volume of radius a (Eqs. (2) and (4) in main text). The data were best fit using $2a$ = 8 and 10 Å at 255 and 265 K, respectively (inset). A similar range of values was estimated independently from fitting the plateau at low-Q in the $\Gamma_T(Q^2)$ plot (panel A) using Eq. 4 in the main text. (**D**) EISF for PTI·H_2O with excess water at 275-305 K. Here the observed behaviour (filled squares) can be modelled by assuming rotation of free H_2O molecules within a sphere with radius 0.98 Å determined by the O-H bond length (solid line). The percentage of mobile protons probed by the QENS experiment (Eq. 4 main text) is shown in the inset as a function of temperature between 255 and 330 K. (**E-F**) D_T and D_{loc} dynamics of PTI treated with an excess of water (~30 wt%; green up-triangles) compared with parameters for bulk H_2O (blue squares - QENS (*43*); light blue diamonds - pulsed-laser-heating (*45*); dark blue stars - NMR (*44*) and viscosimetry (*53*)). Lines are drawn as guides to the eye. (**F**) Residence time map (τ_0; raw data modeled with Eq. 1 main text). (**G**) Rotational relation time map (where $\tau_R = \hbar/\Gamma_{rt}$).

REFERENCES AND NOTES

1. D. L. Gin, R. D. Noble, Designing the next generation of chemical separation membranes. *Science* **332**, 674–676 (2011).

2. E. Tajkhorshid, P. Nollert, M. Ø. Jensen, L. J. W. Miercke, J. O'Connell, R. M. Stroud, K. Schulten, Control of the selectivity of the aquaporin water channel family by global orientational tuning. *Science* **296**, 525–530 (2002).

3. A. Horner, P. Pohl, Single-file transport of water through membrane channels. *Faraday Discuss.* **209**, 9–33 (2018).

4. I. Kocsis, Z. Sun, Y. M. Legrand, M. Barboiu, Artificial water channels—Deconvolution of natural aquaporins through synthetic design. *NPJ Clean Water* **1**, 13 (2018).

5. G. Liu, W. Jin, N. Xu, Graphene-based membranes. *Chem. Soc. Rev.* **44**, 5016–5030 (2015).

6. J. R. Werber, C. O. Osuji, M. Elimelech, Materials for next-generation desalination and water purification membranes. *Nat. Rev. Mater.* **1**, 16018 (2016).

7. A. Lerf, A. Buchsteiner, J. Pieper, S. Schöttl, I. Dekany, T. Szabo, H. P. Boehm, Hydration behavior and dynamics of water molecules in graphite oxide. *J. Phys. Chem. Solid* **67**, 1106–1110 (2006).

8. B. Mi, Graphene oxide membranes for ionic and molecular sieving. *Science* **343**, 740–742 (2014).

9. R. R. Nair, H. A. Wu, P. N. Jayaram, I. V. Grigorieva, A. K. Geim, Unimpeded permeation of water through helium-leak-tight graphene-based membranes. *Science* **335**, 442–444 (2012).

10. J. Muscatello, F. Jaeger, O. K. Matar, E. A. Müller, Optimizing water transport through graphene-based membranes: Insights from nonequilibrium molecular dynamics. *ACS Appl. Mater. Interfaces* **8**, 12330–12336 (2016).

11. S. Jiao, Z. Xu, Non-continuum intercalated water diffusion explains fast permeation through graphene oxide membranes. *ACS Nano* **11**, 11152–11161 (2017).

12. J. Deng, Y. You, H. Bustamante, V. Shajwalla, R. K. Joshi, Mechanism of water transport in graphene oxide laminates. *Chem. Sci.* **8**, 1701–1704 (2017).

13. Y. Wang, N. Wu, Y. Wang, H. Ma, J. Zhang, L. Xu, M. K. Albolkany, B. Liu, Graphite phase carbon nitride based membrane for selective permeation. *Nat. Commun.* **10**, 2500 (2019).

14. R. H. Tunuguntla, R. Y. Henley, Y.-C. Yao, T. A. Pham, M. Wanunu, A. Noy, Enhanced water permeability and tunable ion selectivity in subnanometer carbon nanotube porins. *Science* **357**, 792–796 (2017).

15. Z. Siwy, F. Fornasiero, Improving on aquaporins. *Science* **357**, 753 (2017).

16. A. Horner, P. Pohl, Comment on “Enhanced water permeability and tunable ion selectivity in subnanometer carbon nanotube porins”. *Science* **359**, eaap9173 (2018).

17. R. H. Tunuguntla, Y. Zhang, R. Y. Henley, Y.-C. Yao, T. A. Pham, M. Wanunu, A. Noy, Response to Comment on “Enhanced water permeability and tunable ion selectivity in subnanometer carbon nanotube porins”. *Science* **359**, eaap1241 (2018).

18. E. Wirnhier, M. Döblinger, D. Gunzelmann, J. Senker, B. V. Lotsch, W. Schnick, Poly(triazine imide) with intercalation of lithium and chloride ions $[(C_3N_3)_2(NH_xLi_{1-x})_3 \cdot LiCl]$: A crystalline 2D carbon nitride network. *Chem. A Eur. J.* **17**, 3213–3221 (2011).

19. T. M. Suter, T. S. Miller, J. K. Cockcroft, A. E. Aliev, M. C. Wilding, A. Sella, F. Corà, C. A. Howard, P. F. McMillan, Formation of an ion-free crystalline carbon nitride and its reversible intercalation with ionic species and molecular water. *Chem. Sci.* **10**, 2519–2528 (2019).

20. Z. Zhang, K. Leinenweber, M. Bauer, L. A. J. Garvie, P. F. McMillan, G. H. Wolf, High-pressure bulk synthesis of crystalline $C_6N_9H_3 \cdot HCl$: A novel C_3N_4 graphitic derivative. *J. Am. Chem. Soc.* **123**, 7788–7796 (2001).

21. M. Bée, *Quasielastic Neutron Scattering: Principles and Applications in Solid State Chemistry, Biology and Material Science* (Bristol: Adam Hilger, 1988).

22. F. Volino, A. Dianoux, Neutron incoherent scattering law for diffusion in a potential of spherical symmetry: General formalism and application to diffusion inside a sphere. *Mol. Phys.* **41**, 271–279 (1980).

23. M.-C. Bellissent-Funel, S.-H. Chen, J.-M. Zanotti, Single-particle dynamics of water molecules in confined space. *Phys. Rev. E* **51**, 4558–4569 (1995).

24. G. Hummer, J. C. Rasaiah, J. P. Noworyta, Water conduction through the hydrophobic channel of a carbon nanotube. *Nature* **414**, 188–190 (2001).

25. G. Briganti, G. Rogati, A. Parmentier, M. Maccarini, F. De Luca, Neutron scattering observation of quasi-free rotations of water confined in carbon nanotubes. *Sci. Rep.* **7**, 45021 (2017).

26. E. Mamontov, C. J. Burnham, S.-H. Chen, A. P. Moravsky, C.-K. Loong, N. R. de Souza, A. I. Kolesnikov, Dynamics of water confined in single- and double-wall carbon nanotubes. *J. Chem. Phys.* **124**, 194703 (2006).

27. J. Martí, C. Caler, G. Franzese, Structure and dynamics of water at carbon-based interfaces. *Entropy* **19**, 135 (2017).

28. C. Y. Won, S. Joseph, N. R. Aluru, Effect of quantum partial charges on the structure and dynamics of water in single-walled carbon nanotubes. *J. Chem. Phys.* **125**, 114701 (2006).

29. H. Kyakuno, K. Matsuda, Y. Nakai, R. Ichimura, T. Saito, Y. Miyata, K. Hata, Y. Maniwa, Rotational dynamics and dynamical transition of water inside hydrophobic pores of carbon nanotubes. *Sci. Rep.* **7**, 14834 (2017).

30. L. Lin, T. M. Weigand, M. W. Farthing, P. Jutaporn, C. T. Miller, O. Coronell, Relative importance of geometrical and intrinsic water transport properties of active layers in the water permeability of polyamide thin-film composite membranes. *J. Membr. Sci.* **564**, 935–944 (2018).

31. J. Lyu, X. Wen, U. Kumar, Y. You, V. Chen, R. K. Joshi, Separation and purification using GO and r-GO membranes. *RSC Adv.* **8**, 23130–23151 (2018).

32. Y. Hong, J. Zhang, C. Zhu, X. C. Zeng, J. S. Francisco, Water desalination through rim functionalized carbon nanotubes. *J. Mater. Chem. A* **7**, 3583–3591 (2019).

33. J. B. Heymann, A. Engel, Aquaporins: Phylogeny, structure, and physiology of water channels. *News Physiol. Sci.* **14**, 187–193 (1999).

34. M. J. Bojdys, J. O. Muller, M. Antonietti, A. Thomas, Ionothermal synthesis of crystalline, condensed, graphitic carbon nitride. *Chemistry* **14**, 8177–8182 (2008).

35. V. F. Sears, Theory of cold neutron scattering by homonuclear diatomic liquids: I. Free rotation. *Can. J. Phys.* **44**, 1279–1297 (1966).

36. O. Arnold, J. C. Bilheux, J. M. Borreguero, A. Buts, S. I. Campbell, L. Chapon, M. Doucet, N. Draper, R. Ferraz Leal, M. A. Gigg, V. E. Lynch, A. Markvardsen, D. J. Mikkelson, R. L. Mikkelson, R. Miller, K. Palmen, P. Parker, G. Passos, T. G. Perring, P. F. Peterson, S. Ren, M. A. Reuter, A. T. Savici, J. W. Taylor, R. J. Taylor, R. Tolchenov, W. Zhou, J. Zikovsky, Mantid—Data analysis and visualization package for neutron scattering and μ SR experiments. *Nucl. Instrum. Methods Phys. Res. A* **764**, 156–166 (2014).

37. R. T. Azuah, L. R. Kneller, Y. Qiu, P. L. W. Tregenna-Piggott, C. M. Brown, J. R. D. Copley, R. M. Dimeo, DAVE: A comprehensive software suite for the reduction, visualization, and analysis of low energy neutron spectroscopic data. *J. Res. Natl. Inst. Stand. Technol.* **114**, 341–358 (2009).

38. R. Dovesi, A. Erba, R. Orlando, C. M. Zicovich-Wilson, B. Civalleri, L. Maschio, M. Rérat, S. Casassa, J. Baima, S. Salustro, B. Kirtman, Quantum-mechanical condensed matter simulations with CRYSTAL. *WIREs Comput. Mol. Sci.* **8**, e1360 (2018).

39. J. Hutter, M. Iannuzzi, F. Schiffmann, J. VandeVondele, cp2k: Atomistic simulations of condensed matter systems. *WIREs Comput. Mol. Sci.* **4**, 15–25 (2014).

40. F. Demmel, K. Pokhilchuk, The resolution of the tof-backscattering spectrometer OSIRIS: Monte Carlo simulations and analytical calculations. *Nucl. Instr. Methods Phys. Res. A* **767**, 426–432 (2014).

41. M. Appel, B. Frick, A. Magerl, A flexible high speed pulse chopper system for an inverted neutron time-of-flight option on backscattering spectrometers. *Phys. Rev. B* **562**, 6–8 (2019).

42. A. Mayer, R. M. Dimeo, P. M. Gehring, D. A. Neumann, The high-flux backscattering spectrometer at the NIST Center for Neutron Research. *Rev. Sci. Instrum.* **74**, 2759–2777 (2003).

43. J. Teixeira, M.-C. Bellissent-Funel, S.-H. Chen, A. J. Dianoux, Experimental determination of the nature of diffusive motions of water molecules at low temperatures. *Phys. Rev. A* **31**, 1913–1917 (1985).

44. F. X. Prielmeier, E. W. Lang, R. J. Speedy, H. D. Lüdemann, The pressure dependence of self diffusion in supercooled light and heavy water. *Ber. Bunsen. Phys. Chem* **92**, 1111–1117 (1988).

45. Y. Xu, N. G. Petrik, R. S. Smith, B. D. Kay, G. A. Kimmel, Growth rate of crystalline ice and the diffusivity of supercooled water from 126 to 262 K. *Proc. Natl. Acad. Sci. U.S.A.* **113,** 14921–14925 (2016).

46. J.-C. Perrin, S. Lyonnard, F. Volino, Quasielastic neutron scattering study of water dynamics in hydrated nafion membranes. *J. Chem. Phys.* **111**, 3393–3404 (2007).

47. J. G. Wijmans, R. W. Baker, The solution-diffusion model: A review. *J. Membr. Sci.* **107**, 1–21 (1995).

48. L. A. Perry, O. Coronell, Reliable, bench-top measurements of charge density in the active layers of thin-film composite and nanocomposite membranes using quartz crystal microbalance technology. *J. Membr. Sci.* **429**, 23–33 (2013).

49. L. Lin, R. Lopez, G. Z. Ramon, O. Coronell, Investigating the void structure of the polyamide active layers of thin-film composite membranes. *J. Membr. Sci.* **497**, 365–376 (2016).

50. H. Li, Z. Song, X. Zhang, Y. Huang, S. Li, Y. Mao, H. J. Ploehn, Y. Bao, M. Yu, Ultrathin, molecular-sieving graphene oxide membranes for selective hydrogen separation. *Science* **342**, 95–98 (2013).

51. D. Cohen-Tanugi, J. C. Grossman, Water desalination across nanoporous graphene. *Nano Lett.* **12**, 3602–3608(2012).

52. K. Murata, K. Mitsuoka, T. Hirai, T. Walz, P. Agre, J. B. Heymann, A. Engel, Y. Fujiyoshi, Structural determinants of water permeation through aquaporin-1. *Nature* **407**, 599–605 (2000).

53. L. P. Singh, B. Issenmann, F. Caupin, Pressure dependence of viscosity in supercooled water and a unified approach for thermodynamic and dynamic anomalies of water. *Proc. Natl. Acad. Sci. U.S.A.* **114**, 4312–4317 (2017).
